# Trends in Diatom Research Since 1991 Based on Topic Modeling

**DOI:** 10.3390/microorganisms7080213

**Published:** 2019-07-24

**Authors:** Yun Zhang, Juan Tao, Jun Wang, Liuyong Ding, Chengzhi Ding, Yanling Li, Qichao Zhou, Dunhai Li, Hucai Zhang

**Affiliations:** 1 Institute for Ecological Research and Pollution Control of Plateau Lakes, School of Ecology and Environmental Science, Yunnan University, Kunming 650500, China; 2 Yunnan Key Laboratory of International Rivers and Transboundary Eco-Security, Institute of International Rivers and Eco-Security Yunnan University, Kunming 650500, China; 3 Key Laboratory of Algal Biology, Institute of Hydrobiology, Chinese Academy of Sciences, Wuhan 430072, China

**Keywords:** bibliometrics, cold topic, diatom, hot topic, Latent Dirichlet Allocation

## Abstract

Diatoms are fundamental carbon sources in a wide range of aquatic food webs and have the potential for wide application in addressing environmental change. Understanding the evolution of topics in diatom research will provide a clear and needed guide to strengthen research on diatoms. However, such an overview remains unavailable. In this study, we used Latent Dirichlet Allocation (LDA), a generative model, to identify topics and determine their trends (i.e., cold and hot topics) by analyzing the abstracts of 19,000 publications from the Web of Science that were related to diatoms during 1991–2018. A total of 116 topics were identified from a Bayesian model selection. The hot topics (diversity, environmental indicator, climate change, land use, and water quality) that were identified by LDA indicated that diatoms are increasingly used as indicators to assess water quality and identify modern climate change impacts due to intensive anthropogenic activities. In terms of cold topics (growth rate, culture growth, cell life history, copepod feeding, grazing by microzooplankton, zooplankton predation, and primary productivity) and hot topics (spatial-temporal distribution, morphology, molecular identification, gene expression, and review), we determined that basic studies on diatoms have decreased and that studies tend to be more comprehensive. This study notes that future directions in diatom research will be closely associated with the application of diatoms in environmental management and climate change to cope with environmental challenges, and more comprehensive issues related to diatoms should be considered.

## 1. Introduction

Diatoms (Class Bacillariophyceae) are a group of unicellular, photosynthetic, and eukaryotic algae with silica shells found in almost all types of habitats in freshwater and marine ecosystems [1,2]. Till now, totally more than 100,000 diatom species from over 1250 genera with diverse morphological forms in the world [3]. The large-scale ecological success of diatoms suggests that they have refined their cellular processes for efficient utilization of nutrients and sunlight to a greater extent than most other unicellular algae [2]. Although the individual size of diatoms is generally small (<200 μm), together, they play a vital role in fixing carbon dioxide and synthesizing carbohydrates, contributing to more than 20% of global primary production [4,5]. Most primary and secondary consumers in aquatic ecosystems depend on diatoms to some extent and even exclusively [6,7]. There are also many fish, shrimp and shellfish species of great fishery values that mainly feed on diatoms [8,9,10]. Therefore, diatoms are the main carbon source fueling a wide range of aquatic food webs and supporting a large proportion of global fisheries [11,12].

The global population explosion and accompanying human disturbances since the industrial revolution have resulted in large environmental changes and problems (e.g., climate change and eutrophication) [13]. Diatoms have the potential for wide application in related environmental research and management. First, diatoms can serve as indicators of environmental changes due to their high sensitivity and preservability. For example, diatoms respond to environmental changes (e.g., climate and salinity) by altering individual morphology/size and their assemblages [14,15,16,17,18]. Second, diatoms are a powerful surrogate for reconstructing paleoenvironmental history and even population dynamics of specific aquatic animals [19,20]. The silica shells of diatoms are easily preserved and provide a useful tool for fossil research [21,22,23]. Third, diatoms can also be used to assess environmental health, monitor environmental change [24,25], and then guide environmental management and restoration.

Promoting research on a specific area depends on a clear and objective overview of its development history and current status. However, this kind of overview of diatom research remains unavailable. A research overview can be achieved through a bibliometric analysis [26,27,28], in combination with sophisticated machine learning (ML) technologies (Latent Dirichlet Allocation, LDA) on existing literature [29]. LDA is a flexible generative probabilistic unsupervised topic model used to analyze the changes in topic importance over time for text documents [29]. LDA methods have been applied widely in various fields, including the fields of biology and environmental science, such as biodiversity [30], climate change [31], and animal communities [32]. This study aims to provide an objective overview of diatom research based on publication data from the Web of Science (WoS) during 1991–2018. We use ML technologies to identify research topics and track topic changes over time. This study has two objectives: (1) to summarize the topics in diatom research and (2) to identify the past trends and future directions of diatom research by analyzing the trends in diatom research topics. Hopefully, it will provide insights that will contribute to the future development of diatom research and thus global sustainability.

## 2. Materials and Methods

### 2.1. Data Collection

The data in the present work were sourced from the online database of the Science Citation Index Expanded (SCIE), Web of Science, which is the most comprehensive and frequently used data source in bibliometrics [33]. The exact query for diatom was TS = ((“diatom” OR “diatoms” OR “Bacillariophyta”) NOT (“diatomic” OR “diatomite” OR “diatomaceous earth”)), and the timespan was from 1991 to 2018. The publications in the sub-categories associated with biology, ecology and environmental science, including “agriculture, multidisciplinary”, “biology”, “biodiversity conservation”, “ecology”, “engineering, environmental”, “environmental sciences”, “limnology”, “marine and freshwater biology”, “microbiology”, “oceanography”, “paleontology”, “toxicology”, and “water resources” were selected. Raw data containing titles, keywords, abstracts, year of publication, journal of publication, authors’ names, authors’ affiliations, number of times cited, and cited reference counts were exported. All documents were used to analyze the scientific production per year, distribution in source, and publication distribution by countries. Impact factors (IF) were taken from the Journal Citation Reports (JCR) published in 2018. We used the abstract data from 1991 to 2018 to build the topic models.

### 2.2. Document-Terms Matrix Construction

The input data format for building topic models is a document-terms matrix (DTM), which consists of rows corresponding to the documents (papers) and columns that correspond to the terms (words found in each paper’s abstract). To generate the document-term matrix, raw textual data were pre-processed [34]. Punctuation, numbers, whitespace and stop words in the text and corpus were removed. Words were stemmed, in that they were reduced to their underlying root form, and terms that occurred less than five times were removed. We converted the abstract corpus into a document-term matrix using the function *DocumentTermMatrix* in the ‘tm’ package [35].

### 2.3. Topic Modeling Analysis

Latent Dirichlet Allocation (LDA) is a probabilistic latent variable model of documents that exploit the correlations among the words and latent semantic themes [36]. In LDA, each document in the text corpus is modelled as a set of draws from a mixture distribution over a set of hidden topics, where topics are assumed to be uncorrelated and each topic is characterized by a distribution over words. LDA has been widely used to explore, discover and illustrate key research topics in various academic fields, as well as the rise or fall in topic popularity [29].

**Figure 1:**
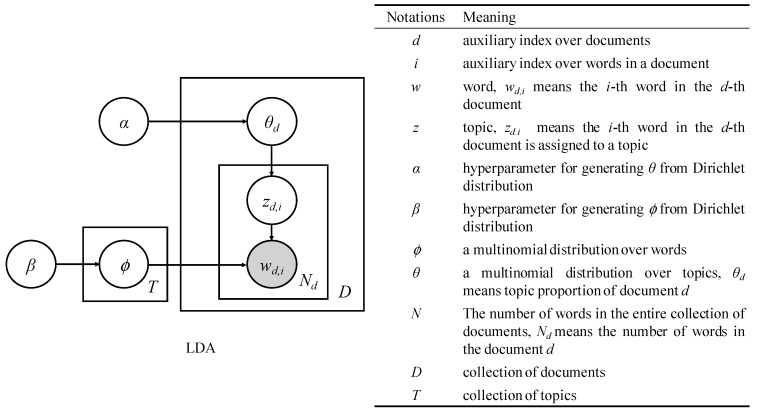
Latent Dirichlet Allocation (LDA) and the meaning of notations (adapted from Ponweiser [39], and Hu et al. [40]).

LDA provides a probabilistic model for the latent topic layer [37] (see Figure 1 for the graphical model representation of LDA). For each document *d*, a multinomial distribution *θ* over topics is sampled from a Dirichlet distribution with parameter α. For each word *w_d_*_,*i*_, a topic *z_d_*_,*i*_ is chosen from a topic-specific multinomial distribution *φ_z_*_,*d*,*i*_ sampled from a Dirichlet distribution with parameter β. The probability of generating a word *w* from a document *d* is:

$$P\left(w|d,\;\theta ,\;\phi \right)=\underset{z\in T}{\sum }P(w|z,\;\phi_{z}{)P(z|d,\;\theta }_{d})$$

Therefore, the likelihood of document collection *D* is defined as:

$$P\left(Z,W|\Theta ,\;\Phi \right)=\underset{d\in D}{\prod }\underset{z\in T}{\prod }\theta_{d,z}^{n_{d,z}}\times \underset{z\in T}{\prod }\underset{v\in V}{\prod }\phi_{z,v}^{n_{z,v}}$$

where *n_d,z_* is the number of times that topic *z* has been associated with document *d*, and *n_z,v_* is the number of times that word *w* has been generated by topic *z*.

*T* represents the number of topics. This study used a statistical method for determining the value of *T*, following the suggestions of Newton and Raftery [38] and Griffiths and Steyvers [29]. The suggestion for determining *T*, referring to a harmonic mean, allows the discovery of the optimal number of topics, as well as a measure of the goodness-of-fit in the modelling. By using the calculation of the harmonic mean, which is one of the maximal log-likelihood methods, the optimum number of topics (*T*) can be ascertained.

### 2.4. Identification of Hot and Cold Topics

The mean *θ* (the per-document probabilities for topics) by year can reflect the rise and fall in the amount of scientific interest in topics. Here, we present a basic analysis based on a post hoc examination of the estimates of *θ* produced by the model. To identify topics that were consistently rising or falling in popularity from 1991 to 2018, we conducted a linear trend analysis on *θ* of each topic by year. According to the slope values (i.e., positive and negative) and their significance levels (i.e., *p* = 0.05, *p* = 0.01, *p* = 0.001, and *p* = 0.0001), topics were identified as “hot” topics and “cold” topics. 

## 3. Results

### 3.1. Characteristic of Publication Outputs

**Figure 2:**
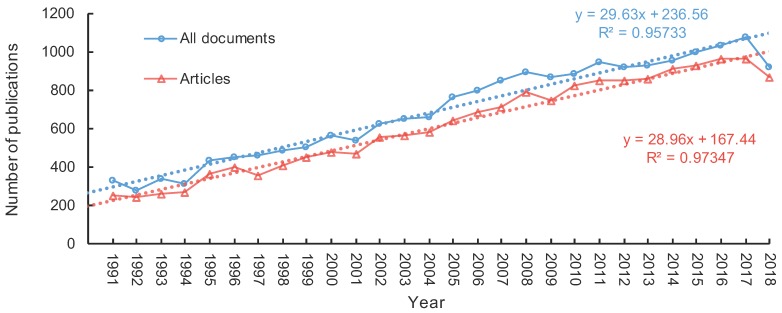
Journal publications with diatom in title, abstract and keywords during 1991–2018 in Web of Science.

A total of 19,483 documents with “diatom/s” or “Bacillariophyta” terms in the title, abstract or keywords were found among the 13 subject categories from 1991 to 2018 (Supplementary E). Article was the most frequent document type, accounting for 88.7% of the total production (Figure 2). Both of the total documents and articles demonstrated a linear increasing tendency in the past three decades, with an annual increase of 29 documents/articles. There were 19,000 documents with abstracts from 1991 to 2018, and we used these documents to identify topics and determine their trends.

The top 20 most active journals on diatom research are listed in Table 1, as well as their total number of publications (TP), total number of publications on diatom (TPD), the ratio of number of publications on diatom with the total number of publications (TPD/TP), total citation counts of publications on diatom (TC), average citation of each publication on diatom from 1991 to 2018 (TC/TPD) and impact factors (IF). The number of articles related to diatom published on a journal is associated with the total number of publications in this journal, therefore, we normalized the data in Table 1. *Diatom research* reasonably had the highest ratio of TPD/TP (80.2%), as it only accepts papers related to diatom, followed by *Journal of Paleolimnology* (34.4%), *Journal of Phycology* (17.1%), *Cryptogamie Algologie* (16.4%), and *European Journal of Phycology* (15.0%). *Limnology and Oceanography* had the highest average citation per article on diatom (62.7), which had a relatively high IF (4.325) among these 20 journals. The following journals with relatively high average citation were *Marine Chemistry* (45.3, IF 2.713)*, Deep-Sea Research Part II-Topical Studies in Oceanography* (44.8, IF 2.430), *Journal of Phycology* (40.3, IF 2.831), and *Protist* (35.2, IF 3.000). The journals with an IF below 2.000 had low average citation according to Table 1.

**Figure 3:**
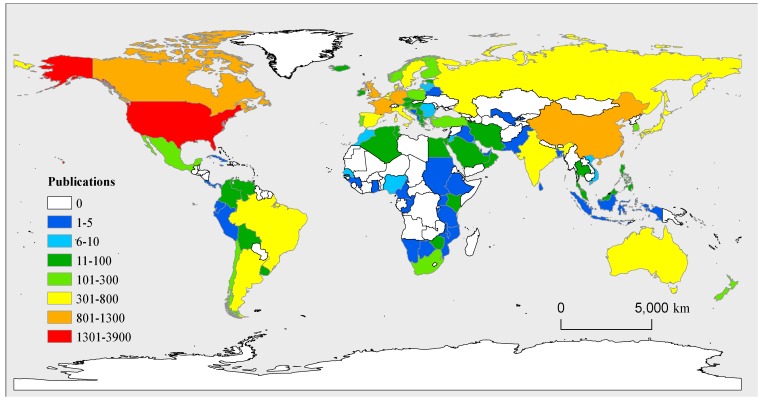
Distribution map of worldwide research on diatom from 1991 to 2018 based on number of publications.

A total of 109 countries/regions have published documents focusing on diatoms in these sub-categories during the period analyzed (Figure 3). The United States (USA) published the most documents related to diatom (3879), followed by Canada (1230), the United Kingdom (1223), France (1149), China (1146), and Germany (1131). The top twelve countries with the most documents published account for 70.73% of the total number of publications. Europe and North America accounted for 45.43% and 28.42% of the total publications, respectively, followed by Asia (15.32%), Oceania (4.86%) and South America (4.24%). Africa accounted for only 1.75% of the total publications.

**Figure 4:**
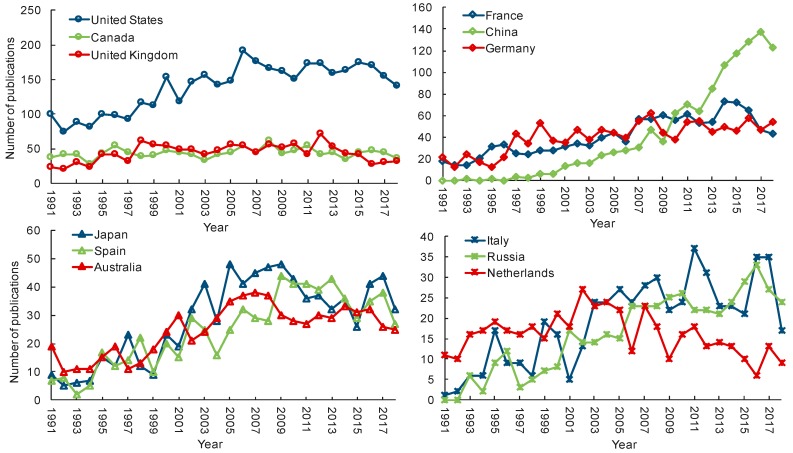
The top 12 countries with most publications related to diatom in title, abstract and keywords during 1991-2018 on Web of Science.

The publication trends of the top 12 countries from 1991 to 2018 were presented in Figure 4. United States, France, China, Germany, Italy, and Russia have an uptrend in publishing documents on diatom since 1991, among which, China has the most obvious increased trend. The number of publications per year in Canada and United Kingdom have not changed much since 1991. Diatom-related publications in Japan, Spain, Australia, and Netherlands had increased from 1991 to the early 21th century, but has decreased in the last decade.

### 3.2. Research Topics and Major Themes in Diatom Research

A total of 116 topics were extracted using the LDA model (Supplementary A Figure S1). The 15 most likely terms and the potential themes of the 116 topics are listed in Supplementary A Table S1. We found 18 uninformative topics according to the extracted terms, and we grouped the remaining 98 topics into four broader themes: *Biology and Life History*, *Biodiversity and Distribution*, *Environmental Changes and Algal Blooms*, and *Applications and Others*; the four broader themes included 17, 32, 35, and 14 topics and 3788, 4193, 7477, and 2791 documents, respectively (Supplementary A Table S2). The broader theme of *Biodiversity and Distribution* is related to the community structure and the spatial or/and temporal distribution of diatoms, including community composition, biodiversity, habitats and temporal variations. *Environmental Changes and Algal Blooms* refers to the environmental pressure (e.g., eutrophication, nutrient limitation, heavy metal, climate change, hydrodynamics, and parasites) on diatoms and the harmful algal blooms caused by environmental changes. *Biology and Life History* is mainly related to the biology and physiology of diatoms, including growth, morphology, photosynthesis, and metabolic products. The broader theme of *Applications and Others* includes the use of diatoms in feeding zooplankton and the larvae of other animals. The other themes mainly involve the study methods, such as remote sensing, stable isotope, microscopic scanning and constructing models.

### 3.3. Hot and Cold Topics

A total of 44 topics were identified as cold topics at the significance level of *p* = 0.05 (Table 2). Among these topics, 17 topics showed a significant decreasing linear trend at the *p* = 0.0001 level (Table 2). Thirty-one of the topics were identified as hot topics at the significance level of *p* = 0.05 level (Table 2), and 17 of the topics showed a significant increasing linear trend at the *p* = 0.0001 level (Table 2).

**Figure 5:**
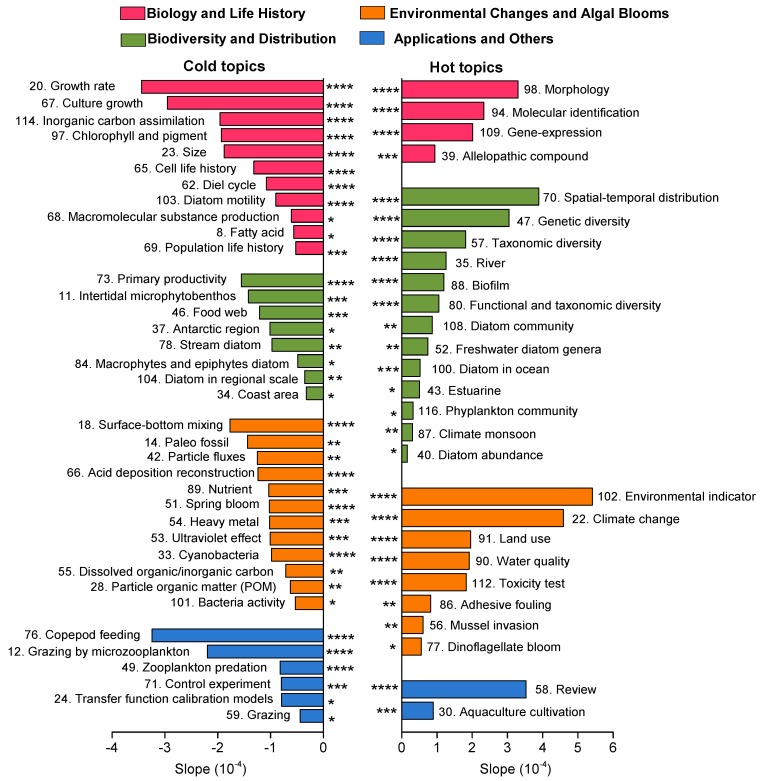
The significant cold and hot topics sorted by the slope of the linear models for the four broader themes. (* represents *p* = 0.05, ** represents *p* = 0.01, *** represents *p* = 0.001, and **** represents *p* = 0.0001).

The slope trend of each topic in the four broader themes is shown in Figure 5. The positive slopes (hot topics) represent the increasing trend in topic popularity, and the negative slopes (cold topics) represent the decreasing trend in topic popularity from 1991 to 2018. In the *Biology and Life History* theme, the top three hot topics are morphology, molecular identification, and gene expression (*p* = 0.0001); the top cold topics include growth rate, culture growth, inorganic carbon assimilation, chlorophyll and pigment, size, cell life of history, diel cycle, and diatom motility (*p* = 0.0001). In the *Biodiversity and Distribution* theme, the hot topics related to spatial-temporal distribution, diversities, river, and biofilm are the most prevalent (*p* = 0.0001); the cold topics of primary productivity (*p* = 0.0001), intertidal microphytobenthos, and food web (*p* = 0.001) decreased most in popularity over time. In the *Environmental Changes and Algal Bloom* theme, the hot topics of environmental indicator, climate change, land use, water quality, and toxicity test are the most prevalent (*p* = 0.0001); cold topics of surface-bottom mixing, acid deposition reconstruction, spring bloom, and cyanobacteria declined extremely significantly over time (*p* = 0.0001). In the *Application and Others* theme, the hot topics of review (*p* = 0.0001) and aquaculture cultivation (*p* = 0.001) are the most prevalent; the cold topics of copepod feeding, grazing by microzooplankton, and zooplankton predation (*p* = 0.0001) became unpopular from 1991 to 2018.

## 4. Discussion

This study objectively summarized research topics and identified the trends in diatom research by using bibliometric analysis and machine learning models for the first time. We found that the documents related to diatoms were largely published in Europe and North America. The LDA model indicated that the popular topics of diversity, environmental indicator, climate change, land use, and water quality signify that diatoms are increasingly used as indicators to assess water quality and identify modern climate change impacts on aquatic ecosystems. From most cold topics (basic topics, such as growth rate, culture growth, cell life history, copepod feeding, grazing by microzooplankton, zooplankton predation, and primary productivity) associated with hot topics (spatial-temporal distribution, morphology, molecular identification, gene expression, and review), we determined that basic studies on diatoms decreased, and studies tended to be more comprehensive. Some other cold topics (surface–bottom mixing and spring bloom) have been well studied in recent decades, and currently, few studies focus on these topics.

Our results reveal that research using diatoms to indicate or assess environmental change has become more popular. With intensive human disturbances, environmental changes such as the degradation of water quality and climate change have become greater concerns of ecologists and environmentalists. Diatoms are the most useful tool for monitoring running waters. They are the key component of river periphytic algae and react over a brief period to changes in water quality. The use of diatoms in bio-assessments was initiated starting in the early 20th century [25], warranted by decreased water quality in streams and rivers induced by anthropogenic activities such as the expansion of developed areas and agricultural areas. Climate change, especially global warming, is another issue of great concern caused by human activities that has a great impact on aquatic ecosystems. Climate warming changed the timing of spring phytoplankton blooms [41] and enhanced the intensity of harmful algae blooms [42], which have led to a significant increase in research on the topic of climate change over the past two decades. We also noted that the studies on paleoclimate (topics Holocene climate, Paleolimnology, acid deposition reconstruction) related to diatoms presented a decreasing trend popularity over these decades (the data are not shown). In paleoclimatic studies, researchers used diatoms to reconstruct ancient climates and learn about the changing climate [43]. However, after intensive studies, researchers determined that climate change is largely associated with modern limnology, and it is a global issue that deserves more attention. Thus, diatoms afford an elevated degree of precision in assessing the trophic status of a water body and reflecting climate change in modern aquatic ecosystems.

Along with the development of science and technology, studies on diatoms are becoming increasingly comprehensive, with decreasing individual basic studies. First, research on the growth and culture of diatoms, as well as feeding on zooplankton, were popular in the “early” days, with broader studies of diatoms in the “future”, and these fields were considered as the basis for further research on diatoms (e.g., species competition studies with batch cultures, aquaculture operations involving seminatural food chains, toxicological tests, and biofuel production). Second, the physiological, morphological and behavioral traits of diatoms (chlorophyll and pigment, inorganic carbon assimilation, diel cycle, diatom motility, and size) have been studied declining trends, and these traits have also been used as the basis for research on hot topic (functional diversity, which involves various traits of diatoms). Third, studies of diatom community structure have expanded at a larger spatio-temporal scale [24,44,45], with more ecologists recognizing that diatoms are more likely to be spatially and temporally structured [46]. Fourth, researchers have simultaneously focused on the multiple facets of biodiversity, such as taxonomic diversity, genetic diversity and functional diversity [47,48], as they have realized that globally decreasing biodiversity resulting from anthropogenic disturbances are predicted to have severe effects on aquatic ecosystem functions [49]. Fifth, with the advent of electronic scanning microscopy and the development of molecular technology, the substructure [50] of diatoms and the sequencing of the 18S rRNA gene for diatoms [51] have been widely used to classify diatoms as opposed to only diatom morphology under light microscopy. Finally, as an increasing number of studies on diatoms have been published, researchers have summarized, analyzed and refined data, materials, and main points in a large number of original research papers and have combined some of their own points of view to write a review related to diatoms. Thus, this the topic of review also presented an increasing trend in popularity over time. 

Some topics related to diatoms had been well studied by previous researchers, so they were less studied by later researchers. For example, the relationship between the cold topic of surface–bottom mixing and diatoms has already been acquainted by most ecologists and biologists. Thermal stratification and vertical mixing (hydrodynamics) cause changes in underwater light conditions and nutrient concentrations and further affect the dynamics of diatoms [52]. This process would lead to the seasonal dynamics of the algae community. The mechanisms of seasonal changes in algae in both fresh and marine waters have been well demonstrated in the Plankton Ecology Group (PEG) model by Sommer et al. [53]. The spring bloom is the first step in the PEG model of phytoplankton succession [53], and in most cases, diatoms occupy the spring blooms. In spring, the water column is well mixed, and the high availability of nutrients enables diatom communities to develop biomass peaks. In addition, the temperature in spring was found to be suitable for the proliferation of many diatom species [54,55,56].

The analysis of country/regional publications indicates that Europe and North America have contributed much more in terms of diatom research than have Oceania, South America, and Africa. One of the possible reasons for this result is official languages. Non-English-speaking countries are hindered in writing and publishing articles in English. Another possible reason is related to development levels of these regions. In undeveloped countries, there are few researchers, and scientific research is inadequately supported by governments, especially in most countries of Africa. We also noticed that the number of publications in China has increased notably since 2009. The same phenomenon also occurred in the studies related to “eutrophication” [57], “nitrogen” [58], and “climate change” [26] using bibliometric analysis. In 2007, the pollution of drinking water caused by cyanobacteria bloom in Lake Taihu led to serious social and economic impacts in China, and the water environment health and protection has received much more attentions. Diatom has increasingly been used as environmental indicator to assess the environmental health, and the number of publications related to diatom therewith increased significantly.

This study also has some potential limitations. For example, this study only provided the first affiliation country’s frequency distribution, but not the exact sites where the studies conducted. In most cases, the first affiliation address is consistent with the study location. However, there are also some inconsistences between them. Therefore, conclusions drawn from using the affiliation country as the surrogate of study area may not accurate enough. Further synthesize studies, particularly those expected to acquire general rules from case studies conducted in gradient geolocations, should extract the exact location of study sites.

## 5. Conclusions

Our study identified 116 topics from the abstracts of 19,000 publications in Web of Science associated with “diatom” using latent Dirichlet allocation (LDA) from 1991 to 2018. We found that 18 of the 116 topics were uninformative and grouped the remaining 98 topics into four broader areas or themes: Biology and Life History, Biodiversity and Distribution, Environmental Changes and Algal Blooms, and Applications and Others. In addition, we have explored the topics that consistently rose (hot topics) and fell (cold topics) in popularity from 1991 to 2018. The degradation of water quality and climate change caused by intensive anthropogenic activities has resulted in the current increase in use of diatoms to indicate water quality and reflect modern climate change. Moreover, with the development of science and technology, basic research on diatoms has decreased, and more studies tend to be comprehensive. The bibliometrics analysis of diatom-related publications indicated that there are more publications produced in developed regions compared with undeveloped regions. This study highlighted that future research directions on diatoms will develop as environmental or climate changes occur and science and technology develop. Diatoms will be widely applied in managing aquatic environments and reflecting climate change to cope with environmental challenges.

## Figures and Tables

**Table 1:** The top 20 most active journals on diatom research from 1991 to 2018.

Journal Name (Category Name)	TPD/TP (%)	TPD	TP	TC	TC/TPD	IF
DIATOM RESEARCH (Marine and Freshwater Biology)	80.2%	483	602	3685	7.6	1.169
JOURNAL OF PALEOLIMNOLOGY (Environmental Sciences; Limnology)	34.4%	601	1747	15,380	25.6	2.009
JOURNAL OF PHYCOLOGY (Marine and Freshwater Biology)	17.1%	774	4524	31,181	40.3	2.831
CRYPTOGAMIE ALGOLOGIE (Marine and Freshwater Biology)	16.4%	116	706	756	6.5	1.361
EUROPEAN JOURNAL OF PHYCOLOGY (Marine and Freshwater Biology)	15.0%	268	1782	5172	19.3	2.526
JOURNAL OF PLANKTON RESEARCH (Marine and Freshwater Biology; Oceanography)	14.9%	506	3404	15,694	31.0	2.209
PHYCOLOGICAL RESEARCH (Marine and Freshwater Biology)	14.3%	76	531	645	8.5	1.342
AQUATIC MICROBIAL ECOLOGY (Ecology; Marine and Freshwater Biology; Microbiology)	14.2%	268	1889	8505	31.7	2.788
HARMFUL ALGAE (Marine and Freshwater Biology)	13.6%	203	1497	4918	24.2	5.012
LIMNOLOGY AND OCEANOGRAPHY (Limnology; Oceanography)	12.9%	729	5635	45,741	62.7	4.325
OCEANOLOGICAL AND HYDROBIOLOGICAL STUDIES (Marine and Freshwater Biology; Oceanography)	12.2%	69	565	276	4.0	0.674
DEEP-SEA RESEARCH PART II-TOPICAL STUDIES IN OCEANOGRAPHY (Oceanography)	11.7%	524	4460	23,456	44.8	2.430
MARINE MICROPALEONTOLOGY (Paleontology)	11.4%	150	1321	3924	26.2	2.663
FUNDAMENTAL AND APPLIED LIMNOLOGY (Limnology; Marine and Freshwater Biology)	11.4%	79	696	639	8.1	0.980
PROTIST (Microbiology)	10.0%	91	910	3202	35.2	3.000
PHYCOLOGIA (Marine and Freshwater Biology)	9.5%	354	3709	6936	19.6	1.976
AQUATIC ECOLOGY (Ecology; Limnology; Marine and Freshwater Biology)	9.5%	76	799	1121	14.8	2.505
VIE ET MILIEU-LIFE AND ENVIRONMENT (Ecology; Marine and Freshwater Biology)	9.2%	65	710	387	6.0	0.362
BIOFOULING (Marine and Freshwater Biology)	8.9%	131	1475	4368	33.3	2.847
MARINE CHEMISTRY (Oceanography)	8.3%	229	2770	10,380	45.3	2.713

TPD total number of publications on diatom research, TP total number of publications in this journal, TC total citation counts of the publications on diatom research, IF impact factor from the Journal Citation Report (JCR) published in 2018.

**Table 2:** Topics with significant negative and positive trends at four significant levels.

Topic Trend	*p* = 0.05	*p* = 0.01	*p* = 0.001	*p* = 0.0001
Negative trend	44	34	27	17
Positive trend	31	27	21	17
Total	75	61	48	34

## Supplementary Materials

The following are available online at https://www.mdpi.com/2076-2607/7/8/213/s1, Supplementary A provided the model selection results, showing the Harmonic mean of the data for different settings of the number of topics (Figure S1); The 15 most probable terms and the potential theme of the 116 topics (Table S1); The topics of the four broader themes and the number of documents in each topic (Table S2). Supplementary B provided the R code of all analysis in this study (RStudio, version 1.2.1226). Supplementary C provided the publications title that are arranged in descending order according to the contribution rate to each topic. Supplementary D and Supplementary E provided downloaded data and filtered data from Web of Science respectively (The data was converted to a format that can be recognized by bibliometric analysis in RStudio using the function of “covert2df”).
