# Supplementary material for: Trends in Diatom Research Since 1991 Based on Topic Modeling
Source: Microorganisms. 2019 Jul 24;7(8):213. doi: 10.3390/microorganisms7080213 (PMC6722707; doi:10.3390/microorganisms7080213)
Supplement: Supplementary file 1 [file microorganisms-07-00213-s001.zip › Supplementary A.doc]

**Supplementary material A**

**Figure S1** Model selection results, showing the Harmonic Mean of the data for different settings of the number of topics (2-200, the optimal number of topics is 116)

**Table** **S1** The 15 most probable terms and the potential theme of the 116 topics

| **Topic** | **Potential theme** | **Top high frequency terms** |
| --- | --- | --- |
| Topic 1 | Uninformative | stations; station; total; period; June; July; august; April; highest; September; March; values; October; sampling; January |
| Topic 2 | Marine bloom seasonality | species; costatum; skeletonema; diatom; Bacillariophyceae; investigated; studied; Chlorophyceae; marinoi; showed; observed; affinis; study; obtained; Dinophyceae |
| Topic 3 | Uninformative | higher; lower; differences; significantly; compared; significant; showed; greater; similar; observed; values; difference; found; times; highest |
| Topic 4 | Uninformative | correlated; relationship; significant; correlation; related; positive; showed; positively; significantly; concentration; found; negative; strong; relationships; negatively |
| Topic 5 | Stable isotope | delta; values; parts; stable; isotope; thousand; isotopic; carbon; isotopes; fractionation; composition; signatures; similar; sources; variations |
| Topic 6 | Macroalgae biofuels | oil; macroalgae; brown; macroalgal; crude; marine; hydrocarbons; examined; kelp; pahs; thalli; results; seaweed; petroleum; aromatic |
| Topic 7 | Silicon requirement | silica; biogenic; BSI; dissolution; diatom; silicon; DSI; acid; silicic; diatoms; concentrations; dissolved; opal; siliceous; frustules |
| Topic 8 | Fatty acid | fatty; acid; acids; lipid; composition; content; total; lipids; amino; omega; EPA; levels; profiles; seston; polyunsaturated |
| Topic 9 | Oceanic upwelling | waters; coastal; upwelling; shelf; offshore; surface; continental; region; oceanic; coast; slope; conditions; current; area; data |
| Topic 10 | Uninformative | study; results; present; potential; order; environment; aim; investigate; determine; show; obtained; main; work; carried; time |
| Topic 11 | Intertidal microphytobenthos | sediment; sediments; benthic; intertidal; microphytobenthos; surface; content; tidal; biomass; erosion; MPB; sand; resuspension; bed; sandy |
| Topic 12 | Grazing by microzooplankton | biomass; grazing; heterotrophic; ciliates; diatoms; microzooplankton; dominated; dinoflagellates; autotrophic; phytoplankton; community; mesozooplankton; flagellates; large; size |
| Topic 13 | Red tide | pseudonitzschia; acid; toxic; species; domoic; toxin; shellfish; detected; pungens; australis; toxins; genus; multiseries; levels; produced |
| Topic 14 | Paleo fossil | sediments; marine; late; deposits; formation; early; middle; upper; age; deposited; miocene; fossil; deposition; pliocene; rise |
| Topic 15 | Uninformative | studies; field; previous; results; laboratory; study; shown; reported; found; suggest; previously; observations; data; show; demonstrated |
| Topic 16 | Diatom assemblage | diatom; assemblages; assemblage; taxa; composition; conditions; dominated; examined; characterized; identified; small; related; dominance; zones; typical |
| Topic 17 | Photosynthesis | photosynthetic; photosynthesis; light; efficiency; fluorescence; irradiance; maximum; parameters; physiological; yield; high; activity; rate; response; showed |
| Topic 18 | Surface-bottom mixing | surface; depth; layer; water; vertical; mixing; mixed; zone; deep; upper; column; layers; depths; distribution; maximum |
| Topic 19 | Microscopic structure | colonies; microscopy; electron; form; process; formed; formation; structures; observed; wall; frustules; scanning; colony; living; mucilage |
| Topic 20 | Growth rate | growth; rates; rate; maximum; day; specific; higher; lower; accumulation; measured; experiments; estimated; determined; dilution; high |
| Topic 21 | Algal mats | mats; microbial; carbonate; diatoms; cyanobacteria; mat; oxygen; microorganisms; precipitation; springs; calcite; anoxic; calcium; formation; conditions |
| Topic 22 | Climate change | change; response; climate; responses; ecosystem; effects; ecosystems; warming; conditions; shifts; PCO; future; global; environmental; levels |
| Topic 23 | Size | size; particles; TEP; aggregates; particle; marine; larger; formation; large; smaller; aggregation; aggregate; small; concentration; sizes |
| Topic 24 | Transfer function calibration models | data; models; transfer; set; based; model; regression; function; diatom; developed; calibration; functions; applied; weighted; averaging |
| Topic 25 | Eutrophication | nutrient; eutrophication; concentrations; phosphorus; loading; water; reduction; quality; total; sewage; increased; eutrophic; treatment; loads; oxygen |
| Topic 26 | Parasites | host; foraminifera; viruses; reef; coral; hosts; parasite; fungal; fungi; infection; parasites; virus; viral; reefs; populations |
| Topic 27 | Sea ice melting | ice; sea; arctic; antarctica; cover; seaice; antarctic; ross; shelf; bottom; surface; polar; fragilariopsis; greenland; melting |
| Topic 28 | Particle Organic Matter (POM) | organic; matter; particulate; carbon; material; suspended; total; sources; degradation; sediments; terrestrial; POM; origin; marine; derived |
| Topic 29 | Uninformative | sites; site; sampling; differences; study; collected; locations; located; sampled; compared; clear; related; difference; unique; base |
| Topic 30 | Aquaculture cultivation | microalgae; microalgal; potential; conditions; aquaculture; cultivation; evaluated; energy; biomass; chlorella; effective; significant; storage; tetraselmis; fresh |
| Topic 31 | Uninformative | system; systems; open; frequency; compared; presented; studied; terms; made; closed; controlled; discussed; frequencies; remaining; allowing |
| Topic 32 | Paleolimnology | lake; lakes; sediment; shallow; recent; years; diatom; trophic; change; assemblages; past; paleolimnological; sediments; state; Chironomid |
| Topic 33 | Cyanobacteria | algal; algae; green; alga; filamentous; biomass; bluegreen; cyanobacteria; found; studied; scenedesmus; chlamydomonas; chlorophytes; chlorella; amount |
| Topic 34 | Coast area | bay; channel; fjord; coastal; inlet; USA; outer; harbour; bays; sound; florida; regions; columbia; occurred; british |
| Topic 35 | River | river; water; flow; rivers; discharge; downstream; upstream; hydrological; main; riverine; high; large; flood; upper; channel |
| Topic 36 | Uninformative | order; magnitude; diatoms; percentage; characteristics; quantitative; qualitative; studied; comparison; determined; selection; orders; made; presented; degree |
| Topic 37 | Antarctic region | southern; antarctic; region; waters; south; ocean; zone; front; surface; polar; distribution; north; transect; northern; frontal |
| Topic 38 | Uninformative | hypothesis; support; results; evidence; test; oysters; supported; oyster; suggest; findings; significant; tested; hypotheses; supports; consistent |
| Topic 39 | Allelopathic compound | compounds; marine; activity; extracts; chemical; diatom; metabolites; produce; effect; allelopathic; tested; extract; produced; compound; potential |
| Topic 40 | Diatom abundance | abundance; relative; abundances; diatoms; abundant; total; highest; study; distribution; high; numbers; absolute; dominant; composition; analyzed |
| Topic 41 | Uninformative | samples; collected; found; sample; analysis; analyzed; determined; analysed; study; identified; included; analyses; salmon; present; revealed |
| Topic 42 | Particle fluxes | flux; fluxes; carbon; export; sinking; POC; particulate; sediment; organic; material; particles; trap; traps; particle; depth |
| Topic 43 | Estuarine | salinity; estuary; freshwater; estuarine; lagoon; coastal; tidal; marine; estuaries; salt; brackish; marsh; salinities; zone; saline |
| Topic 44 | Eddy and cyclone | similar; eddy; eddies; center; large; mesoscale; times; cyclonic; approximate; evident; DOT; greater; variable; features; anticyclonic |
| Topic 45 | Iron limitation | iron; phytoplankton; ocean; limitation; concentrations; patch; waters; availability; fertilization; diatoms; ratios; HNLC; dissolved; fold; inside |
| Topic 46 | Food web | food; feeding; trophic; diet; web; contents; prey; diatoms; gut; detritus; webs; energy; important; source; ingested |
| Topic 47 | Genetic diversity | diversity; community; analysis; communities; rRNA; microbial; sequences; detected; marine; DNA; revealed; eukaryotic; sequencing; gene; diverse |
| Topic 48 | Uninformative | elsevier; reserved; rights; science; published; study; based; significant; research; copyright; due; crown; GMBH; main; results |
| Topic 49 | Zooplankton predation | zooplankton; plankton; large; daphnia; pelagic; small; rotifers; Copepods; Cladocerans; planktonic; dominated; predation; Cladoceran; phyto; ecosystem |
| Topic 50 | Global distribution | ocean; north; pacific; atlantic; region; surface; regions; equatorial; western; eastern; global; subarctic; subtropical; dust; gyre |
| Topic 51 | Spring bloom | bloom; blooms; phytoplankton; spring; phaeocystis; development; early; diatom; observed; occurred; peak; dynamics; dominated; period; late |
| Topic 52 | Freshwater diatom genera | diatom; taxa; species; nitzschia; navicula; var; diatoms; cocconeis; achnanthes; gomphonema; amphora; fragilaria; found; genera; achnanthidium |
| Topic 53 | Ultraviolet | radiation; UVR; exposed; par; solar; exposure; effects; UVB; active; enhanced; ultraviolet; effect; damage; photosynthetically; UVBR |
| Topic 54 | Heavy metal | concentrations; metal; metals; concentration; trace; accumulation; elements; copper; cadmium; zinc; heavy; seawater; dissolved; contamination; ligands |
| Topic 55 | Dissolved organic/Inorganic carbon | dissolved; carbon; organic; doc; inorganic; release; huxleyi; concentrations; DOM; total; pool; particulate; seawater; pools; emiliania |
| Topic 56 | Mussel invasion | geminata; mussels; great; mussel; native; zealand; association; invasive; freshwater; didymosphenia; spread; introduced; invasion; potential; international |
| Topic 57 | Taxonomic diversity | species; diversity; taxa; richness; number; found; composition; identified; genera; recorded; dominant; belonging; studied; taxonomic; biodiversity |
| Topic 58 | Review | studies; research; ecological; information; environmental; understanding; provide; future; recent; ecology; data; organisms; knowledge; including; review |
| Topic 59 | Grazing | effects; grazing; effect; interactions; density; densities; grazers; direct; reduced; influence; results; control; affect; competition; grazer |
| Topic 60 | Uninformative | time; period; days; day; periods; short; end; occurred; recovery; series; long; times; rapid; longer; observed |
| Topic 61 | Holocene climate | core; sediment; lake; record; holocene; climate; conditions; years; cal; records; pollen; cores; glacial; period; change |
| Topic 62 | Light effect | light; conditions; mol; irradiance; intensity; dark; low; high; intensities; diel; darkness; photons; regimes; cycle; irradiances |
| Topic 63 | Larvae Feeding | larvae; larval; survival; settlement; abalone; fed; growth; juvenile; length; shrimp; diet; juveniles; metamorphosis; postlarvae; day |
| Topic 64 | Reservoir bloom | cyanobacteria; reservoir; cyanobacterial; reservoirs; microcystis; water; aeruginosa; diatoms; blooms; eutrophic; cyanobacterium; anabaena; dominant; filamentous; aphanizomenon |
| Topic 65 | Cell life history | cells; cell; density; size; volume; diatom; division; densities; number; content; initial; numbers; live; cellular; death |
| Topic 66 | Acid deposition reconstruction | deposition; acidification; soil; acidic; atmospheric; chemistry; chemical; soils; acid; critical; acidified; recovery; biological; diatom; peat |
| Topic 67 | Culture growth | growth; cultures; culture; phase; medium; grown; marine; cell; cells; cellular; conditions; thalassiosira; content; weissflogii; tricornutum |
| Topic 68 | Macromolecular substance production | eps; fraction; extracellular; carbohydrates; fractions; carbohydrate; substances; colloidal; polysaccharides; polymeric; soluble; molecular; glucose; proteins; produced |
| Topic 69 | Population life history | population; populations; stages; resting; stage; life; cycle; spores; conditions; development; vegetative; formation; bank; spore; viable |
| Topic 70 | Spatial-temporal distribution | spatial; patterns; environmental; variability; temporal; variation; scales; distribution; structure; local; scale; community; factors; regional; communities |
| Topic 71 | Control experiment | experiments; experiment; treatments; addition; increased; control; treatment; experimental; natural; conducted; added; significantly; effect; effects; results |
| Topic 72 | Freshwater diatom species | species; cyclotella; diatom; aulacoseira; stephanodiscus; diatoms; formosa; fragilaria; dominant; asterionella; granulata; meneghiniana; baikal; hantzschii; freshwater |
| Topic 73 | Primary productivity | production; primary; productivity; net; carbon; biomass; rates; high; estimated; measured; respiration; oxygen; gross; mmol; measurements |
| Topic 74 | Marine diatom genera | spp; species; chaetoceros; diatoms; thalassiosira; diatom; coscinodiscus; genera; waters; coastal; rhizosolenia; study; centric; nitzschia; distribution |
| Topic 75 | Remote sensing | data; absorption; measurements; chlorophyll; situ; properties; fluorescence; optical; spectra; satellite; spectral; variability; remote; measured; concentration |
| Topic 76 | Copepod feeding | copepods; copepod; food; feeding; egg; production; rates; fed; ingestion; females; eggs; success; pellets; diet; prey |
| Topic 77 | Dinoflagellate bloom | dinoflagellate; dinoflagellates; species; harmful; red; blooms; cysts; prorocentrum; alexandrium; algal; minimum; akashiwo; brevis; tides; potentially |
| Topic 78 | Stream diatom | stream; periphyton; streams; biomass; benthic; algal; communities; diatoms; epilithic; habitat; composition; community; structure; invertebrate; substrata |
| Topic 79 | Uninformative | presence; observed; present; absence; closterium; found; cylindrotheca; revealed; addition; investigated; diatom; detected; absent; general; examined |
| Topic 80 | Functional and taxonomic diversity | groups; group; functional; type; taxonomic; types; diatoms; based; major; distinct; analysis; differences; ecological; traits; level |
| Topic 81 | Uninformative | important; role; play; factors; processes; factor; key; dynamics; study; importance; controlling; major; results; understanding; influence |
| Topic 82 | Seasonal variation | summer; spring; seasonal; winter; year; autumn; annual; years; late; period; early; variations; variability; seasons; pattern |
| Topic 83 | Storm effect | events; sand; sponge; storm; tsunami; sponges; including; wave; deposits; event; storms; spicules; marine; deposit; due |
| Topic 84 | Macrophytes and epiphytes diatom | fish; epiphytic; plant; plants; macrophytes; macrophyte; submerged; seagrass; vegetation; habitat; aquatic; diatoms; epiphytes; leaves; fishes |
| Topic 85 | Water masses | water; column; ballast; bottom; study; oxygen; exchange; masses; characteristics; due; mass; caused; potential; including; pressure |
| Topic 86 | Adhesive fouling | surface; surfaces; removal; fouling; marine; adhesion; organisms; coatings; antifouling; attachment; settlement; biofilm; process; removed; biofouling |
| Topic 87 | Climate monsoon | coast; monsoon; study; waters; west; forms; arabian; found; scintillans; noctiluca; southwest; coastal; northeast; india; east |
| Topic 88 | Biofilm | communities; community; biofilms; biofilm; composition; structure; natural; artificial; colonization; development; periphytic; succession; substrates; study; density |
| Topic 89 | Nutrient | nitrate; nitrogen; phosphate; ammonium; silicate; concentrations; source; urea; concentration; nitrite; uptake; ammonia; sources; mol; nutrients |
| Topic 90 | Water quality | analysis; environmental; variables; factors; total; showed; water; conductivity; correspondence; chemical; distribution; data; analyses; variation; significant |
| Topic 91 | Human activities/ agriculture, land use | land; human; activities; wetlands; aquatic; impacts; watershed; wetland; disturbance; natural; anthropogenic; agricultural; management; ecosystem; catchment |
| Topic 92 | Benthic, planktonic and pelagic diatom | diatoms; diatom; benthic; planktonic; pennate; centric; pelagic; suggests; large; determine; forms; additionally; lack; examined; fact |
| Topic 93 | Nutrient limitation | nutrient; nutrients; nitrogen; limitation; phosphorus; concentrations; ratios; ratio; inorganic; limited; availability; enrichment; conditions; growth; supply |
| Topic 94 | Molecular identification | strains; genetic; morphological; molecular; isolated; species; phylogenetic; rDNA; sequences; isolates; sexual; analyses; sequence; reproduction; data |
| Topic 95 | Variation at dry and wet season | dry; season; tropical; weight; wet; temperate; seasons; brazil; study; rainy; state; analyzed; mass; present; rainfall |
| Topic 96 | Temperature effect | degrees; temperature; temperatures; thermal; sst; degreesc; higher; air; observed; conditions; warmer; warm; cold; compared; range |
| Topic 97 | Chlorophyll and pigment | chl; chlorophyll; pigment; concentrations; pigments; diatoms; phytoplankton; fucoxanthin; concentration; total; chi; hplc; ratios; mug; liquid |
| Topic 98 | Morphology | species; valve; genus; nov; electron; microscopy; morphological; valves; scanning; light; morphology; taxa; features; taxon; shape |
| Topic 99 | Uninformative | method; methods; analysis; samples; techniques; technique; applied; results; identification; standard; based; sample; developed; detection; approach |
| Topic 100 | Diatom in ocean | sea; northern; mediterranean; baltic; china; adriatic; waters; coastal; east; distribution; bering; black; eastern; north; japan |
| Topic 101 | Bacteria activity | bacteria; bacterial; activity; microbial; activities; heterotrophic; attached; enzyme; showed; bacterium; freeliving; strain; isolated; algicidal; substrate |
| Topic 102 | Environmental indicator | quality; index; water; ecological; indices; diatom; status; reference; biological; assessment; pollution; sites; based; monitoring; metrics |
| Topic 103 | Diatom motility | current; velocity; flow; turbulence; conditions; wind; diatoms; motile; speed; direction; motility; movement; effect; behaviour; patches |
| Topic 104 | Diatom in regional scale | area; basin; areas; part; central; western; studied; eastern; basins; located; main; northern; considered; small; entire |
| Topic 105 | Uninformative | range; wide; found; common; reported; large; variety; highly; occur; variable; generally; environments; occurring; show; ranges |
| Topic 106 | Nitrogen fixation | fixation; trichodesmium; unicellular; associations; oligotrophic; evidence; tropical; similar; observed; association; nitrogen; fixed; diazotrophic; newly; detected |
| Topic 107 | Diatom in Mexico California | gulf; mexico; california; studied; provided; blue; ostrearia; respect; forms; haslea; coast; peninsula; marennine; records; baja |
| Topic 108 | Diatom community | phytoplankton; biomass; community; composition; diatoms; dominated; structure; abundance; dinoflagellates; total; dominance; dominant; chlorophyll; small; communities |
| Topic 109 | Gene-expression | genes; protein; gene; expression; proteins; metabolism; involved; tricornutum; genome; sequence; molecular; metabolic; expressed; sequences; enzyme |
| Topic 110 | Prediction model | model; data; models; results; ecosystem; dynamics; observations; processes; developed; simulated; simple; parameters; biological; predicted; simulations |
| Topic 111 | Island habitat | island; ponds; pond; islands; habitats; freshwater; flora; antarctica; archipelago; terrestrial; habitat; east; cove; small; antarctic |
| Topic 112 | Toxicity test | exposure; effects; toxicity; concentrations; exposed; sensitive; effect; test; marine; concentration; toxic; tolerance; sensitivity; stress; copper |
| Topic 113 | Uninformative | increased; increase; decreased; decrease; increasing; concentration; increases; caused; due; resulted; change; reduced; decreasing; reduction; significant |
| Topic 114 | Inorganic carbon assimilation | uptake; assimilation; rates; transport; rate; concentration; carbon; kinetics; activity; experiments; measured; inorganic; constant; internal; external |
| Topic 115 | Uninformative | high; low; levels; level; conditions; concentrations; lower; higher; characterized; extremely; moderate; highest; generally; intermediate; resulted |
| Topic 116 | Phytoplankton community | bacillariophyta; dmsp; chlorophyta; concentrations; dms; cyanophyta; nmol; investigated; cryptophyta; euglenophyta; dinophyta; sulfur; divisions; dimethylsulfoniopropionate; variations |

**Table S2** The topics of the four broader themes and the number of documents in each topic

| **Broader themes** | **Topic number** | **Topics** | **Number of documents** |
| --- | --- | --- | --- |
| Biology and Life History | 98 | Morphology | 698 |
|  | 94 | Molecular identification | 305 |
|  | 109 | Gene-expression | 328 |
|  | 39 | Allelopathic compound | 180 |
|  | 7 | Silicon requirement | 246 |
|  | 17 | Photosynthesis | 288 |
|  | 69 | Population life history | 46 |
|  | 8 | Fatty acid | 305 |
|  | 68 | Macromolecular substance production | 191 |
|  | 103 | Diatom motility | 54 |
|  | 62 | Diel cycle | 80 |
|  | 65 | Cell life history | 65 |
|  | 23 | Size | 194 |
|  | 97 | Chlorophyll and pigment | 252 |
|  | 114 | Inorganic carbon assimilation | 145 |
|  | 67 | Culture growth | 272 |
|  | 20 | Growth rate | 139 |
|  | **Total** |  | **3788** |
| Biodiversity and Distribution | 70 | Spatial-temporal distribution | 237 |
|  | 47 | Genetic diversity | 262 |
|  | 57 | Taxonomic diversity | 134 |
|  | 35 | River | 182 |
|  | 88 | Biofilm | 140 |
|  | 80 | Functional and taxonomic diversity | 40 |
|  | 108 | Diatom community | 294 |
|  | 52 | Freshwater diatom genera | 280 |
|  | 100 | Diatom in ocean | 65 |
|  | 43 | Estuarine | 121 |
|  | 116 | Phytoplankton community | 74 |
|  | 87 | Climate monsoon | 36 |
|  | 50 | Global distribution | 112 |
|  | 40 | Diatom abundance | 8 |
|  | 74 | Marine diatom genera | 101 |
|  | 95 | Variation at dry and wet season | 20 |
|  | 16 | Diatom assemblage | 46 |
|  | 107 | Diatom in Mexico California | 35 |
|  | 111 | Island habitat | 58 |
|  | 92 | Benthic, planktonic and pelagic diatom | 16 |
|  | 2 | Marine bloom seasonality | 38 |
|  | 21 | Algal mats | 145 |
|  | 72 | Freshwater diatom species | 73 |
|  | 34 | Coast area | 44 |
|  | 104 | Diatom in regional scale | 15 |
|  | 84 | Macrophytes and epiphytes diatom | 105 |
|  | 82 | Seasonal variation | 253 |
|  | 78 | Stream diatom | 228 |
|  | 37 | Antarctic region | 214 |
|  | 46 | Food web | 344 |
|  | 11 | Intertidal microphytobenthos | 340 |
|  | 73 | Primary productivity | 133 |
|  | **Total** |  | **4193** |
| Environmental Changes and Algal Blooms | 102 | Environmental indicator | 499 |
|  | 22 | Climate change | 236 |
|  | 91 | Human activities/ agriculture, land use | 127 |
|  | 90 | Water quality | 257 |
|  | 112 | Toxicity test | 343 |
|  | 86 | Adhesive fouling | 181 |
|  | 61 | Holocene climate | 877 |
|  | 56 | Mussel invasion | 91 |
|  | 77 | Dinoflagellate bloom | 188 |
|  | 26 | Parasites | 144 |
|  | 64 | Reservoir bloom | 115 |
|  | 106 | Nitrogen fixation | 70 |
|  | 83 | Storm effect | 36 |
|  | 13 | Red tide | 283 |
|  | 96 | Temperature effect | 46 |
|  | 44 | Eddy and cyclone | 28 |
|  | 25 | Eutrophication | 177 |
|  | 85 | Water masses | 46 |
|  | 9 | Oceanic upwelling | 269 |
|  | 45 | Iron limitation | 213 |
|  | 93 | Nutrient limitation | 188 |
|  | 32 | Paleolimnology | 589 |
|  | 27 | Sea ice melting | 266 |
|  | 101 | Bacteria activity | 139 |
|  | 28 | Particle organic matter | 133 |
|  | 55 | Dissolved organic/inorganic carbon | 124 |
|  | 33 | Cyanobacteria | 51 |
|  | 53 | Ultraviolet effect | 161 |
|  | 54 | Heavy metal | 307 |
|  | 51 | Spring bloom | 142 |
|  | 89 | Nutrient | 95 |
|  | 66 | Acid deposition reconstruction | 113 |
|  | 42 | Particle fluxes | 303 |
|  | 14 | Paleo fossil | 421 |
|  | 18 | Surface-bottom mixing | 219 |
|  | **Total** |  | **7477** |
| Applications and Others | 58 | Review | 275 |
|  | 30 | Aquaculture cultivation | 67 |
|  | 5 | Stable isotope | 133 |
|  | 110 | Prediction model | 240 |
|  | 6 | Macroalgae biofuels | 72 |
|  | 75 | Remote sensing | 145 |
|  | 59 | Grazing | 104 |
|  | 19 | Microscopic structure | 164 |
|  | 24 | Transfer function calibration models | 233 |
|  | 71 | Control experiment | 225 |
|  | 49 | Zooplankton predation | 61 |
|  | 63 | Larvae feeding | 234 |
|  | 12 | Grazing by microzooplankton | 390 |
|  | 76 | Copepod feeding | 448 |
|  | **Total** |  | **2791** |
