# Supplementary material for: Trends in Diatom Research Since 1991 Based on Topic Modeling
Source: Microorganisms. 2019 Jul 24;7(8):213. doi: 10.3390/microorganisms7080213 (PMC6722707; doi:10.3390/microorganisms7080213)
Supplement: Supplementary file 1 [file microorganisms-07-00213-s001.zip › Supplementary B.doc]

**Supplementary material B**

#Code 1 The bibliometrix analysis

library(bibliometrix)

documents<-read.csv('documents.csv', row.names = 1, header=TRUE, stringsAsFactors = TRUE)

documents[,1:24] <- lapply(documents[,1:24],as.character)

results <- biblioAnalysis(documents, sep = ";")

summary(results, k = 50, pause = FALSE)

write.csv(results$Countries, file="Countries.csv") #export the affiliation countries' frequency distribution

write.csv(results$Sources,file="Sources.csv") # export the frequency distribution of sources (journals, books, etc.)

#Code 2 The Latent Dirichlet Allocation (LDA) analysis

library(tm)

library (lattice)

library(topicmodels)

documents<-read.csv('documents.csv', row.names = 1, header=TRUE, stringsAsFactors = TRUE)

docs <- documents$AB

stopwords <- c(tm::stopwords("english"), tm::stopwords("SMART"))

corp <- tm::Corpus(tm::VectorSource(docs))

corp <- tm::tm_map(corp, tm::content_transformer(tolower))

corp <- tm::tm_map(x=corp, tm::removeWords, stopwords)

corp <- tm::tm_map(corp, tm::removePunctuation)

corp <- tm::tm_map(corp, tm::removeNumbers)

corp <- tm::tm_map(corp, tm::stripWhitespace)

dtm <- tm::DocumentTermMatrix(corp)

dtm <- DocumentTermMatrix(corp,

control = list(

tolower = TRUE,

removePunctuation = TRUE,

removeNumbers= TRUE,

stemming = FALSE,

stopwords = TRUE,

minWordLength = 2))

#search the number of topics, which will take a lot of time

library(psych)

seqk<-seq(2,200,1)

burnin<-1000

iter<-1000

keep<-50

system.time(fitted_many_ab<-lapply(seqk, function(k)

topicmodels::LDA(dtm,k=k,method = "Gibbs",control = list(burnin=burnin,

iter=iter,keep=keep))))

save(fitted_many_ab, file = "fitted_many_abstract.RData")

#extract logliks from each topic

logLiks_many<-lapply(fitted_many_ab,function(L) L@logLiks[-c(1:(burnin/keep))])

#compute harmonic means

hm_many<-sapply(logLiks_many,function(h) harmonic.mean(h))

#LDA analysis

load("fitted_many_abstract.RData")

system.time(model_lda <- LDA(dtm, 116, method = "Gibbs",

control = list(iter = 2000, seed = 33)))

save(model_lda, file = "model_lda_abstract.RData")

#export the 15 most likely terms for the 116 topics

load("model_lda_abstract.RData")

terms_15<-terms(model_lda,15)

terms_15<-apply(terms_15,MARGIN = 2,paste,collapse=";")

write.csv(terms_15,file ="topics_terms_15.csv")

#hot and cold topics

topic_most_likely<-topics(model_lda,1)

topics_years<-data.frame(data=(documents$PY),topic_most_likely)

years <- levels(factor(documents$PY))

topics_n <- model_lda@k

theta <- posterior(model_lda)$topics

theta_mean_by_year_by <- by(theta, documents$PY, colMeans)

theta_mean_by_year <- do.call("rbind",theta_mean_by_year_by)

colnames(theta_mean_by_year) = paste(1:topics_n)

theta_mean_by_year_ts <- ts(theta_mean_by_year,

start = as.integer(years[1]),end = as.integer(years[28]))

theta_mean_by_year_time <- time(theta_mean_by_year)

theta_mean_lm <- apply(theta_mean_by_year, 2,

function(x) lm(x ~ theta_mean_by_year_time))

theta_mean_lm_coef <- lapply(theta_mean_lm,

function(x) coef(summary(x)))

theta_mean_lm_coef_sign <- sapply(theta_mean_lm_coef,

'[',"theta_mean_by_year_time","Pr(>|t|)")

theta_mean_lm_coef_slope <- sapply(theta_mean_lm_coef,

'[',"theta_mean_by_year_time","Estimate"]

write.csv(theta_mean_lm_coef_slope, file = "slope.csv")

theta_mean_lm_coef_slope_pos <-

theta_mean_lm_coef_slope[theta_mean_lm_coef_slope >= 0]

theta_mean_lm_coef_slope_neg<-

theta_mean_lm_coef_slope[theta_mean_lm_coef_slope < 0]

p_level <- c(0.05, 0.01, 0.001, 0.0001)

significance_total <- sapply(p_level,

function(x) (theta_mean_lm_coef_sign[theta_mean_lm_coef_sign < x]))

significance_neg <- sapply(1:length(p_level),

function(x) intersect(names(theta_mean_lm_coef_slope_neg),

names(significance_total[[x]])))

significance_pos <- sapply(1:length(p_level),

function(x) intersect(names(theta_mean_lm_coef_slope_pos),

names(significance_total[[x]])))

topics_cold <- as.numeric(names(sort(theta_mean_lm_coef_slope[

significance_neg[[1]]], decreasing=FALSE)))

topics_hot <- as.numeric(names(sort(theta_mean_lm_coef_slope[

significance_pos[[1]]], decreasing=TRUE)))
